# The Spectrum of Beta-Thalassemia Mutations in Syrian Refugees and Turkish Citizens

**DOI:** 10.7759/cureus.15434

**Published:** 2021-06-04

**Authors:** Ahmet Kursad Gunes, Hilmi Erdem Gozden

**Affiliations:** 1 Department of Hematology, Ankara City Hospital, Ankara, TUR; 2 Department of Hematology, Sanliurfa Mehmet Akif Inan Training and Research Hospital, Sanliurfa, TUR; 3 Department of Hematology, Abdulhamid Han Education and Research Hospital, Istanbul, TUR

**Keywords:** beta thalassemia major, beta globin gene mutations, sanliurfa, syrian refugees, turkey

## Abstract

Introduction and objectives

Neighboring the border between Turkey and Syria, Sanliurfa is one of the Turkish provinces with the highest number of Syrian refugees in our country. We aimed to find out the spectrum of beta-globin gene mutations in adult Turkish citizens and Syrian refugees with beta-thalassemia major.

Results

Of the participants, 35 patients (70%) were Turkish citizens and 15 patients (30%) were Syrian. The most common mutation in Turkish patients was found to be IVS-I-110 (G>A) with a frequency of 28.8%, followed by IVS-I-6 (T>C) with a frequency of 15.5%. Other common mutations were IVS-I-1 (G>A) and codon 39 (C>T) with frequencies of 11.1%. These four mutations accounted for 65.5% of all mutations in the Turkish cohort. The most common mutations in Syrian refugee patients were IVS-I-1 (G>A), IVS-II-1 (G>A), IVS-I-5 (G>C), and codon 5 (-CT), all with a frequency of 15.7%, accounting for 62.8% of all mutations in the Syrian patients. In the analysis, codon 5 (-CT) mutation (15.7% vs 0%, p=0.023) was found significantly higher in Syrian refugees compared to Turkish citizens.

Discussion and conclusions

A wide spectrum of mutations was detected in beta-thalassemia major patients living in the Sanliurfa region. Mutational profiles in Turkish and Syrian patients were found to be significantly different from each other. Because marriages between Syrian refugees and Turkish citizens are increasing in our region, the genetic findings and the mutational profiles in Turkish and Syrian patients obtained in this study are thought to become useful for future prenatal molecular diagnostic tests.

## Introduction

Beta thalassemia is a disease resulting from the reduced or absent synthesis of the beta-globin chain in the hemoglobin tetramer because of a genetic defect. The inheritance pattern is an autosomal recessive trait in beta-thalassemia, which is the most common “genetic” hematological disease [1]. As a result of the reduced or absent synthesis of the beta chain, unbound alpha chains precipitate in erythroid precursors, resulting in ineffective erythropoiesis and eventual hemolysis [2]. The clinical and hematological spectrum in beta-thalassemia ranges from an asymptomatic or silent carrier state or beta-thalassemia trait manifested as mild anemia and microcytosis to a prominent clinical picture of beta-thalassemia intermedia or beta-thalassemia major [3].

The beta-globin gene is located on the short arm (11p15.4) of chromosome 11. It is composed of 146 amino acids and three exons. More than 300 beta-thalassemia gene mutations have been identified to date [4]. Unlike alpha thalassemia characterized by large deletions, most beta-thalassemia mutations are point mutations such as a single nucleotide substitution or deletions of one or two nucleotides. However, deletions and insertions are rare in beta-thalassemia [5, 6].

As for the regional distribution of beta-thalassemia, the disease is more commonly seen in the Mediterranean area, the Middle East, Africa, and East Asia [7]. The examination of different ethnic populations and geographical regions reveals significant diversities across the mutational spectra or profiles of the beta-thalassemia gene [8].

The frequency of beta-thalassemia trait is around 2% in Turkey, but such rates may vary by the geographical region [9]. Based on the data from the hemoglobinopathy control program carried out by the Turkish National Hemoglobinopathy Council, the frequency was found as 4.3% in our country [10]. In Sanliurfa, the frequency of beta-thalassemia was reported as 2.44% in a study. This figure is considered to be higher than the general average frequency in Turkey [11]. It has been reported that the high frequency of beta-thalassemia in the Southeast Anatolia region of Turkey is associated with the high rates of consanguineous marriages and high birth rates [12].

Due to the civil war in Syria, 3-3.5 million Syrian citizens have immigrated to Turkey in recent years. Located on the border of Turkey and Syria, Sanliurfa is one of the provinces with the highest number of Syrian refugees in our country [13]. The Republic of Turkey has issued a temporary protection status for Syrian immigrants in our country. Thus, healthcare services and medicine are provided free of charge for all registered refugees in Turkey [13]. In this regard, both Turkish citizens and Syrian refugees with beta-thalassemia major are followed and treated in our center.

This study was conducted aiming to find out beta-globin gene mutations in adult Turkish citizens and Syrian refugees with beta-thalassemia major treated in our center.

## Materials and methods

In our study, 50 Turkish citizens and Syrian refugees with beta-thalassemia major who were treated in our center between the years 2016 and 2019 were analyzed retrospectively. Adult patients older than 18 years of age who were (1) followed up and treated in our center (2) had complete follow-up data (3) underwent genetic analysis, (4) were previously treated in pediatric centers with a diagnosis of beta-thalassemia major, and (5) continued their treatment in our center after reaching adulthood were included in the study. For beta-thalassemia mutation analyses, blood samples were collected in 5 ml EDTA tubes. The collected samples were analyzed in the genetic laboratory. After the amplification of the encoding exons and the exon-intron junctions of the beta globulin gene by the polymerase chain reaction (PCR) method, a DNA sequence analysis was performed. Our study was approved by the Ethics Committee of the Mersin Toros University on 28.04.2020 with the decision number of 28. Written consent was obtained from all patients for genetic diagnosis. Written consent was obtained from Syrian patients in the presence of a sworn translator.

Statistical assessment was performed using SPSS 23 for Windows (IBM SPSS Inc., Armonk, NY, USA). The Kolmogorov-Smirnov test was used to assess whether the data fit a normal distribution. Categorical variables are demonstrated as numbers and percentages. The distribution of numerical variables in two groups was evaluated with an independent samples t-test (numerical variables that fit a normal distribution) or the Mann-Whitney U-test (numerical variables that did not fit a normal distribution). Comparisons of categorical variables in groups were tested with Fisher exact chi-square tests. Values of p<0.05 were recognized to be significant in statistical analyses.

## Results

In this study, 50 patients were studied and a total of 64 mutations in 15 different types were identified. Of the patients, 26 were men (52%) and 24 (48%) were women. The mean age of the patients was 24.1 years and the median age was 22 (18-47) years. Of the patients, 35 (70%) were Turkish citizens and 15 (30%) were Syrian. Homozygous mutations and compound heterozygous mutations were found in 36 (72%) and 14 (28%) patients, respectively. There was no difference between the mean ages of Turkish (23.4 years) and Syrian patients (24.3 years) (p=0.725). While 28.6% of Turkish patients (11 patients) had a compound heterozygous mutation, 27% of Syrian patients (four patients) were found to have a compound heterozygous mutation. There was not a significant difference in the distribution of homozygous/compound heterozygous mutations between the two groups (p=0.891). 

When the mutations of all patients in our region were examined, it was found that the most frequently observed mutation was IVS-I-110 (G> A) with a frequency of 23.4%. IVS-I-6 (T>C) and IVS-I-1 (G>A) (12.5%) were identified as the second- and third-most common mutations. IVS-II-1 (G>A) and codon 39 (C>T) were identified as the fourth- and fifth-most common mutations both with frequencies of 9.4%. These five types of mutations accounted for 67.2% of all mutations. Homozygous and heterozygous mutations and respective frequencies detected in the study patients are summarized in Table 1.

**Table 1:** Distribution and frequencies of 64 mutated alleles of the beta-globin gene in all patients

Mutation	HUGO Nomenclature	Homozygous	Compound Heterozygous	n=64 (%)
IVS-I-110 (G>A)	c.93-21G>A	11	4	15 (23.4%)
IVS-I-6 (T>C)	c.92 + 6T> C	2	6	8 (12.5%)
IVS-I-1 (G>A)	c.92 + 1G>A	6	2	8 (12.5%)
IVS-II-1 (G>A)	c.315 + 1G>A	3	3	6 (9.4%)
Codon 39 (C>T)	c.118C>T	4	2	6 (9.4%)
IVS-I-5 (G>C)	c.92 + 5G>C	1	3	4 (6.2%)
Codon 8 (-AA)	c.25-26delAA	2	1	3 (4.7%)
Codon 5 (-CT)	c.17-18delCT	2	1	3 (4.7%)
Codon 27 (G>T)	c.82G>T	0	2	2 (3.1%)
Codon 6 (-A)	c.20delA	1	1	2 (3.1%)
Codon 44 (-C)	c.135del-C	2	0	2 (3.1%)
Codon 8/9 (+G)	c.27-28insG	1	1	2 (3.1%)
Codon 9/10 (+T)	c.30-31insT	0	1	1 (1.6%)
Codon 15 (G>A)	c.47G>A	0	1	1 (1.6%)
Codon 17 (A>T)	c.52A>T	1	0	1 (1.6%)
Total		36	28	64

In 14 patients, 12 different genotypes of compound heterozygous mutations were detected. Of such mutations, the most common were IVS-I-1 (G>A) and IVS I-5 (G>C) compound heterozygous genotypes identified in three patients. The genotypes of the patients with compound heterozygous mutations are summarized in Table 2.

**Table 2:** The distribution of compound heterozygous beta-thalassemia mutations in all patients

Mutation -1	Mutation -2	N (%)	
IVS-I-1 (G>A)	IVS I-5 (G>C)	3	2 Syrian, 1 Turkish
IVS-I-1 (G>A)	IVS-I-6 (T>C)	1	Turkish
IVS-I-1 (G>A)	Codon 39 (C>T)	1	Turkish
IVS-I-1 (G>A)	IVS II-1 (G>A)	1	Turkish
IVS I-110 (G>A)	Codon 39 (C>T)	1	Turkish
IVS I-110 (G>A)	Codon 6 (A>T)	1	Syrian
IVS I-110 (G>A)	Codon 8/9 (+G)	1	Turkish
IVS I-110 (G>A)	IVS-I-6 (T>C)	1	Turkish
Codon 9/10 (+T)	Codon 15 (G>A)	1	Turkish
Codon 8 del (-AA)	Codon 28 (G>T)	1	Turkish
Codon 27 (G>T)	IVS II-1 (G>A)	1	Turkish
Codon 5 (-CT)	IVS II-1 (G>A)	1	Syrian

A total of 45 mutations of 13 different types were observed in 35 Turkish patients. A total of 19 mutations of nine different types were observed in 15 Syrian patients. The most common mutation in Turkish patients was found to be IVS-I-110 (G>A) with a frequency of 28.8%. The second-most common mutation in Turkish patients was found as IVS-I-6 (T>C) with a frequency of 15.5%. Other common mutations were IVS-I-1 (G>A) and codon 39 (C>T) with frequencies of 11.1%. These four mutations accounted for 65.5% of all mutations.

The most common mutations in Syrian refugee patients were IVS-I-1 (G>A), IVS-II-1 (G>A), IVS-I-5 (G>C), and codon 5 (-CT), all with a frequency of 15.7%. These four mutations accounted for 62.8% of all mutations.

In the analysis, codon 5 (-CT) mutation was significantly higher in Syrian refugees compared to Turkish citizens (15.7% vs 0%, p=0.023). The mutations in Turkish and Syrian patients and the comparison of the groups are summarized in Table 3.

**Table 3:** The mutations in Turkish and Syrian patients and the comparison of both groups

Mutation	HUGO Nomenclature	Turkish Citizen	Syrian refugee	P-value
IVS-I-110 (G>A)	c.93-21G>A	13 (28.8%)	2 (10.4%)	0.176
IVS-I-6 (T>C)	c.92 + 6T> C	7 (15.5%)	1 (5.2%)	0.407
IVS-I-1 (G>A)	c.92 + 1G>A	5 (11.1%)	3 (15.7%)	0.683
IVS-II-1 (G>A)	c.315 + 1G>A	3 (6.6%)	3 (15.7%)	0.348
Codon 39 (C>T)	c.118C>T	5 (11.1%)	1 (5.2%)	0.654
IVS-I-5 (G>C)	c.92 + 5G>C	1 (2.2%)	3 (15.7%)	0.075
Codon 8 (-AA)	c.25-26delAA	3 (6.6%)	-	0.545
Codon 5 (-CT)	c.17-18delCT	-	3 (15.7%)	0.023
Codon 27 (G>T)	c.82G>T	2 (4.4%)	-	0.514
Codon 6 (-A)	c.20delA	1 (2.2%)	1 (5.2%)	0.407
Codon 44 (-C)	c.135del-C	-	2 (10.4%)	0.086
Codon 8/9 (+G)	c.27-28insG	2 (4.4%)	-	0.514
Codon 9/10 (+T)	c.30-31insT	1 (2.2%)	-	1.000
Codon 15 (G>A)	c.47G>A	1 (2.2%)	-	1.000
Codon 17 (A>T)	c.52A>T	1 (2.2%)	-	1.000
Total		45	19	

## Discussion

Sanliurfa is located in Southeast Turkey and it is located near the border of Turkey and Syria. Sanliurfa ranks the third province of Turkey concerning the number of pediatric beta-thalassemia major cases reported. Furthermore, a study on pediatric patients reported that Sanliurfa had the highest rates of consanguineous marriages with a frequency of almost 75% in the parents of beta-thalassemia major patients [12, 14].

In the Turkish registry study, the most common beta-globin chain mutation in pediatric beta-thalassemia major patients in Turkey was found out to be IVS-I-110 (G>A) with a frequency of 47.1%. The second most common mutation was IVS-I-1 (G>A) and the third most frequent mutation was IVS-I-6 (T>C) with frequencies of 7.6% and 7.5%, respectively [12]. In our study, the most common mutation was IVS-I-110 (G>A) with a frequency of 23.1% across all patients included in the study. The frequency of the IVS-I-110 (G>A) mutation was found as 28.8% in Turkish patients. The second- and third-most common mutations in Turkish patients were IVS-I-6 (T>C) and IVS-I-1 (G>A) with frequencies of 15.5% and 11.1%, respectively. The three most common mutations in Turkish patients in our study were found similar to those reported by the aforementioned study but the frequencies were different among the studies. As reported by previous studies, types and frequencies of mutations may show regional variability across the country probably because of ethnic differences. In Turkey, the IVS-I-110 mutation is observed with frequencies of almost 50% in the west, in the Marmara and the Aegean regions of Turkey. However, the frequency of the IVS-I-110 mutation has been shown to be as low as 25-30% in the Eastern and Southeastern Anatolia regions of Turkey [5]. Just as detected in our study, the mutational profile in Syrian patients in our province is significantly divergent compared to the profile reported by the Turkish pediatric registry study. The mutations in Syrian refugee patients were IVS-I-1 (G>A), IVS-II-1 (G>A), IVS-I-5 (G>C), and codon 5 (-CT), each observed with a frequency of 15.7%. In the Turkish registry study, the frequencies of these mutations were found as 7.6%, 5%, 3%, and 2.1%, respectively.

In a study analyzing beta-globin chain mutations in the pediatric population of Turkish citizens in the Sanliurfa region, Ayçiçek et al. found that IVS-I-110 (G>A) was the most common mutation with a frequency of 29%. The second most common mutation was found as IVS-I-1 (G>A) with a frequency of 13.9%. The third most common mutation was codon 39 (C>T) with a frequency of 10.4% [14]. In our study, concordant to the findings of that article, the frequencies of the IVS-I-110, IVS-I-1, and codon 39 (C>T) mutations in Turkish patients were found in frequencies of 28.8%, 11.1%, and 11.1%, respectively. However, the frequencies of the IVS-I-6 mutation in the adult and pediatric populations were 15.5% and 2.2%, respectively.

In a study conducted in Gaziantep, another border city neighboring Sanliurfa, Ulasli et al. found that IVS-I-110 (G>A) was the most frequent mutation in the beta-globin chain with a frequency of 30.6%. The second most common mutation was IVS-I-1 (G>A) with a frequency of 12.4% and the third most common was IVS-I-6 (T>C) with a frequency of 9.4%. The mutation profile in Turkish patients in our city is similar to the profile found out in the neighboring province Gaziantep [15].

In a study conducted in Diyarbakir, another border city neighboring Sanliurfa, Ince et al. found out that the most frequent mutation was IVS-I-110 (G>A) with a frequency of 27.8% [16]. The second-most common was IVS-I-6 (T>C) and the third-most common mutation was codon 8 (-AA) both with a frequency of 11.1%. The frequency of the IVS-I-1 (G>A) mutation is 2.5%. The frequency of codon 8 (-AA) in Turkish citizens in our province has been found as 6.6%. It is noted that the frequencies of the IVS-I-1 (G>A) mutation in Gaziantep and Sanliurfa are higher compared to the frequency in Diyarbakır.

Syria is a Middle Eastern country bordering Turkey. Due to the civil war in Syria, there are approximately 3.5 million refugees in Turkey, who were registered in the context of the temporary protection regime. Syria is one of the regions where thalassemia is common. In an analysis in 2014, investigating the mutation profiles of Syrian patients, the most common mutation was detected as IVS-I-110 (G>A) with a frequency of 17% in 189 patients [17]. It was noted that IVS-I-110 (G>A) is the most common mutation in Syria as in our country. IVS-I-110 (G>A) is the most common mutation in Lebanon [18], Egypt [19], and Greece [20]. The second-most common mutation is IVS-I-1 (G>A) with a frequency of 14.7%. These mutations are followed by codon 39 (C>T) with a frequency of 14.4%, IVS-II-I (G>A) with a frequency of 9.8%, codon 8 (-AA) with a frequency of 6.2%, IVS-I-6 (T>C) with a frequency of 5.2%, IVS-I-5 with a frequency of 4.9%, and codon 5 (-CT) with a frequency of 3.2%. While 75% of beta-thalassemia major patients have a homozygous mutation, 25% have a compound heterozygous mutation [17]. In our study, the most common mutations found in Syrian patients were IVS-I-1 (G>A), IVS-II-1 (G>A), IVS-I-5 (G>C), and codon 5 (-CT), all with a 15.7% frequency.

The IVS-I-1 (G>A) mutation was found as 14.7% in Syrian patients in this analysis but it was reported as 17% in another study on Syrian patients [21]. The IVS-I-1 (G>A) mutation has been observed with a frequency of about 15% in Lebanon [18]. Across European countries, the IVS-I-1 (G>A) mutation is observed with frequencies of 50% in the Czech Republic (1740317) and around 30% in Spain [22]. In Turkish patients in our center, the IVS-I-1 (G>A) mutation was detected at a frequency of 11.1%.

Another mutation, IVS-II-1 (G>A), is the most common one in Arab countries, especially in Erbil - Northern Iraq [23] and Iran [24]. In our study, the frequency of mutations in Syrian patients was found to be 15.7%. It is one of the frequently detected mutations in Saudi Arabia and Kuwait [17].

In a study in 2018 about the geographical distribution of beta-thalassemia mutations in Syria, Murad et al. reported that IVS-I-1 (G> A) was the most frequent mutation in Syria with a frequency of 22.2% [2]. The frequencies of IVS-I-1 (G>A) and IVS-II-1 were found as 17.8% and 7.8%, respectively [2]. In our study, codon 5 (-CT), one of the most common mutations in Syrian patients, was observed with a frequency of 5.6%. In a regional analysis, it was reported that the most frequent mutation was IVS-I-5 (G>C) (22.5%) especially in the northern part of Syria neighboring Sanliurfa (in the cities Aleppo, Idlib, and Raqqa). Other common mutations in that region were reported as IVS-I-1 (G>A) with a frequency of 13.6%, codon 5 (-CT) with a frequency of 12.5%, and IVS-II-1 (G>A) with a frequency of 10%. In our study, contrary to the reports for overall Syria, the mutational profile was found similar to that of Northern Syria. We thought that the mutational profile in our study was found similar to the profile described for that region because the refugees, who had migrated to Sanliurfa, were predominantly from these three cities in Northern Syria bordering our province [2].

## Conclusions

In conclusion, a wide spectrum of mutations was detected in beta-thalassemia major patients living in the Sanliurfa region. Mutational profiles in Turkish and Syrian patients were found to be significantly different from each other. The mutational profiles of Syrian patients were particularly similar to those in Northern Syria. The mutational profile in Turkish patients was akin to the profile described for patients living in the Southeast region of Turkey.

Our study is the first to compare mutational profiles in Turkish and Syrian refugees with thalassemia. In the prevention of genetically inherited diseases such as hemoglobinopathies, genetic counseling and prenatal diagnosis is of particular importance in high-risk geographical regions. Because marriages between Syrian refugees and Turkish citizens are increasing in our region, the genetic findings and the mutational profiles in Turkish and Syrian patients obtained in this study are thought to become useful for future prenatal molecular diagnostic tests.
